# Intravesical protrusion of giant median prostatic lobe: A case report

**DOI:** 10.1016/j.eucr.2022.102152

**Published:** 2022-07-04

**Authors:** M. Duijn, M.C. Hovius, L.M.S. Boevé

**Affiliations:** Department of Urology, OLVG, PO Box 95500, 1090 HM, Amsterdam, the Netherlands

**Keywords:** Benign prostatic hyperplasia, Prostate, Intravesical prostatic protrusion, Lower urinary tract symptoms

## Abstract

Benign prostatic hyperplasia (BPH) is a common disease in ageing men and the result of unregulated hyperplastic growth of the epithelial and stromal tissues of the prostate. Intravesical prostatic protrusion (IPP) of the median lobe is a phenomenon of overgrowth of the prostate adenoma into the bladder and associated with an increased risk for lower urinary tract symptoms (LUTS). This article reports an unusual case of severe intravesical protrusion of a giant median prostatic lobe in 85 years old male with progressive LUTS.

## 1 Introduction

Benign enlargement of the prostate, commonly known as benign prostatic hyperplasia (BPH), is the result of unregulated hyperplastic growth of the epithelial and stromal tissues of the different lobes. Worldwide the lifetime prevalence of BPH is 26.2%.^1^ 50% of men over the age of 50 and up to 80% of men over the age of 80 experience lower urinary tract symptoms (LUTS) affected by BPH.^2^ Progressive LUTS may be increased by intravesical prostatic protrusion (IPP) of the median lobe, a phenomenon of overgrowth of the prostate adenoma into the bladder along the plane of least resistance. We report a case of BPH with severe IPP in an 85-year-old male with progressive LUTS.

## 2 Case presentation

**Fig. 1:**
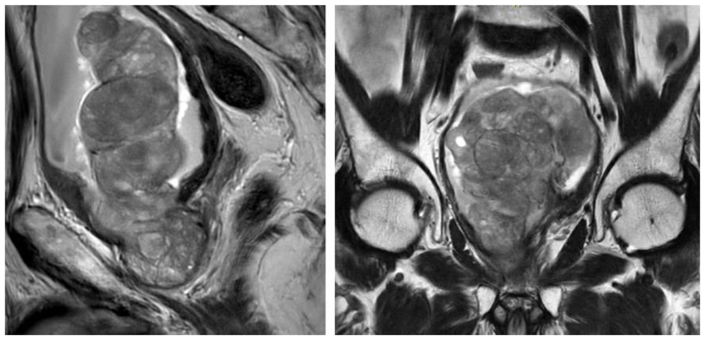
MRI shows intravesical protrusion of giant median prostatic lobe.

An 85-year-old male presented to the outpatient clinic with progressive LUTS including frequency, nocturia, and a weak urine flow. Past medical history was remarkable for multiple transurethral resections of the prostate (TURP). However, these procedures were only partially effective and LUTS returned after a short period of time. Digital rectal examination (DRE) and previous transrectal ultrasonography (TRUS) showed an enlarged prostate, the rest of the physical examination was without abnormalities. Cystoscopy revealed enlarged prostatic lateral lobes and a gigantic prostatic median lobe on the ventral side with severe intravesical protrusion. No urothelial abnormalities were found. PSA was 11 ng/ml. An MRI of the prostate was performed, revealing significantly median lobe enlargement, occupying almost half of the urinary bladder (Fig. 1). The images showed a PIRADS 2 lesion, which means that there was no suspicion of malignant proliferation, and a prostate volume of 109 cc.^3^

**Fig. 2:**
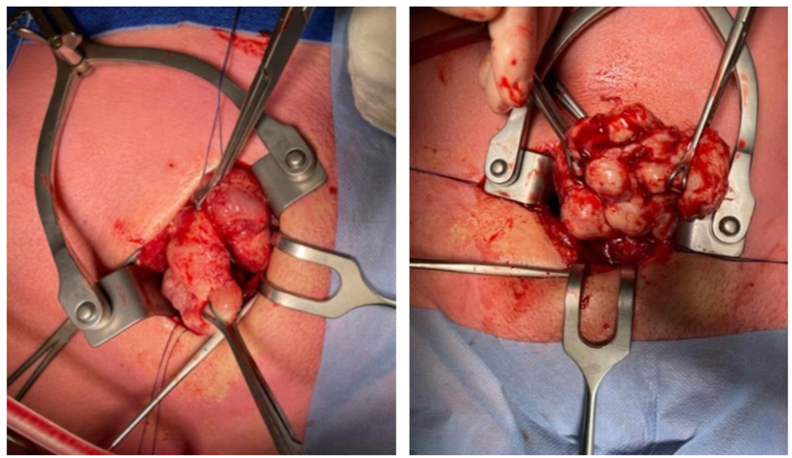
Images of the adenoma during surgery.

**Fig. 3:**
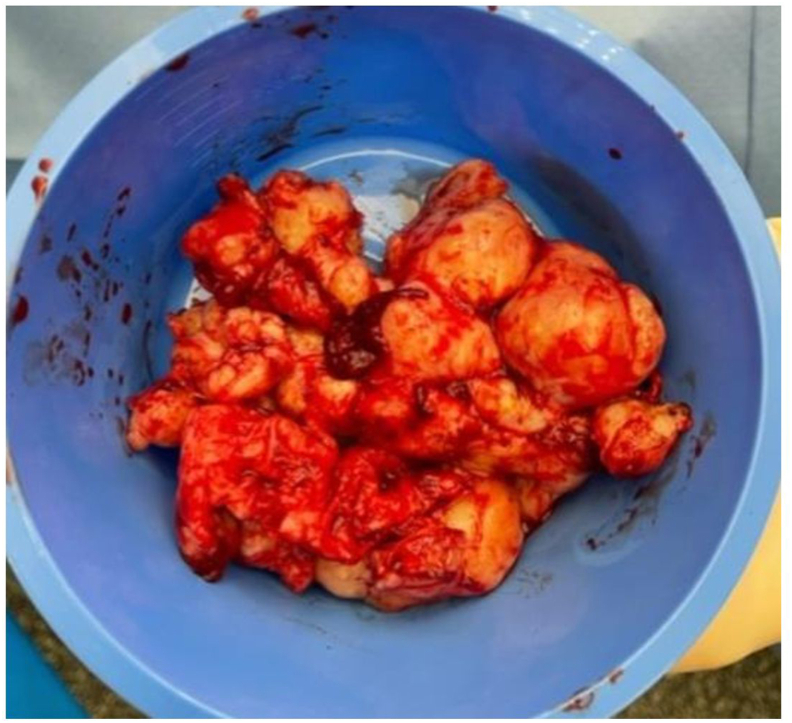
The whole adenoma after its removal.

After obtaining consent, surgery through a transvesical approach was performed, allowing the removal of ten multinodular and lobular tissue fragments, measuring 9.0 cm in totally and weighing 160 g (Fig. 2, Fig. 3). Per-operative a three-way Foley catheter with continuous irrigation system was inserted and a retropubic drain was placed.

We sent the specimen to pathology. Microscopic examination showed benign epithelial hyperplasia of the prostate. No evidence of carcinoma or nephrogenic adenoma was found.

Postoperative, the retropubic drain was removed on the second day. On the fifth day after surgery the three-way Foley catheter was removed. Because of residual urine a two-way indwelling catheter was replaced, and the patient was discharged. After one week the patient showed successful voiding after trial without catheter and presented significant less obstructive symptoms.

## 3 Discussion

BPH is one of the most frequent diseases in ageing men. Its prevalence increases almost linearly over the years to 80% in men older than 80 years.^1^ The prevalence of moderate/severe (IPSS >7) BPH-related LUTS is around 20% in the 5th, 30% in the 6th and 40% in the 8th decade of life.^2^ A common cause of obstructive symptoms including hesitancy, dribbling, straining, prolonged micturition and intermittent urine stream is enlargement of the median prostatic lobe. In addition, Luo et al. demonstrated even an increased rate of bladder outlet obstruction (BOO) or progression of clinical BPH in men with intravesical protrusion of the median lobe. IPP can be graded as grade I (<5 mm), grade II (5–10 mm) and grade III (>10 mm). Severe intravesical protrusion may be associated with a higher risk for (progressive) LUTS.^4^ These findings suggest the clinical importance of adequate diagnosis and treatment of symptomatic BPH with IPP.

Complete surgical removal of the adenoma is suggested the standard therapy of BPH with high grade IPP. In our case we performed a transvesical, open prostatectomy. Low grade IPP can be treated adequately by alpha blockers, 5-alpha reductase inhibitors, TURP or laser enucleation of the prostate.

Despite its high prevalence, the pathophysiology and growth pattern of BPH is still not entirely clear. However, previous studies ascribed the development of BPH to a multifactorial process including the androgen pathway, age-related tissue remodeling, metabolic factors and growth factors/inflammation.^5^

Severe intravesical protrusion of giant median prostatic lobe should be treated adequately by complete surgical resection of the whole adenoma. If not, progressive LUTS will develop. This can have a major impact on patients’ quality of life.

Our patient showed elevated post voiding residual volumes after removal of the urinary catheter. After one week he voided adequately without catheter and with significant less obstructive LUTS. Postoperative inadequate micturition occurs in some patients. However, the majority will eventually void successfully.

## 4 Conclusion

Enlargement of the median prostatic lobe, in the context of BPH, is observed very commonly in aging men and either symptomatic or asymptomatic. On the other hand, giant median lobe enlargement of the prostate is considered a rare case. Complete surgical resection of the prostate is suggested to perform in order to reduce the risk of progressive LUTS.

## Consent

Informed consent was obtained from the patient for publication of this case report in accordance with the journals patient consent policy.

## Funding statement

No funding to declare.

## Ethical approval

The study conforms to recognized standards of World Medical Association Declaration of Helsinki.

## Data availability

The data used and/or analyzed ruing the current study are available from the corresponding authors per request.

## Author contributions

M. Duijn: Conceptualization, Methodology, Writing - original draft preparation, Resources. L.M.S. Boevé: Investigation, Visualization, Writing - review and editing. M.C. Hovius: Writing - review and editing, Validation, Supervision.

## Declaration of competing interest

The authors declare no conflict of interest.
